# Structure–Activity Relationship of Benzofuran Derivatives with Potential Anticancer Activity

**DOI:** 10.3390/cancers14092196

**Published:** 2022-04-28

**Authors:** Joviana Farhat, Lara Alzyoud, Mohammad Alwahsh, Basem Al-Omari

**Affiliations:** 1 Department of Epidemiology and Population Health, College of Medicine and Health Sciences, Khalifa University, Abu Dhabi P.O. Box 127788, United Arab Emirates; joviana.farhat@ku.ac.ae; 2 College of Pharmacy, Al Ain University, Abu Dhabi P.O. Box 64141, United Arab Emirates; lara.alzyoud@aau.ac.ae; 3 Health and Biomedical Research Center, Al Ain University, Abu Dhabi P.O. Box 64141, United Arab Emirates; 4 Leibniz-Institut Für Analytische Wissenschaften-ISAS e.V., 44139 Dortmund, Germany; m.alwahsh@zuj.edu.jo; 5 Institute of Pathology and Medical Research Center (ZMF), University Medical Center Mannheim, Heid Elberg University, 68167 Mannheim, Germany; 6 Department of Pharmacy, Faculty of Pharmacy, AlZaytoonah University of Jordan, P.O. Box 130, Amman 11733, Jordan; 7 KU Research and Data Intelligence Support Center (RDISC) AW 8474000331, Khalifa University of Science and Technology, Abu Dhabi P.O. Box 127788, United Arab Emirates

**Keywords:** benzofuran, SAR, hybrid benzofurans, anticancer activity, anticancer potency, anticancer selectivity

## Abstract

**Simple Summary:**

Cancer is the leading cause of death worldwide and responsible for killing approximately 10 million people per year. Fused heterocyclic ring systems such as benzofuran have emerged as important scaffolds with many biological properties. Furthermore, derivatives of benzofurans demonstrate a wide range of biological and pharmacological activities, including anticancer properties. The main aim of this review is to highlight and discuss the contribution of benzofuran derivatives as anticancer agents by considering and discussing the chemical structure of 20 different compounds. Evaluating the chemical structure of these compounds will guide future medicinal chemists in designing new drugs for cancer therapy that might give excellent results in in vivo/in vitro applications.

**Abstract:**

Benzofuran is a heterocyclic compound found naturally in plants and it can also be obtained through synthetic reactions. Multiple physicochemical characteristics and versatile features distinguish benzofuran, and its chemical structure is composed of fused benzene and furan rings. Benzofuran derivatives are essential compounds that hold vital biological activities to design novel therapies with enhanced efficacy compared to conventional treatments. Therefore, medicinal chemists used its core to synthesize new derivatives that can be applied to a variety of disorders. Benzofuran exhibited potential effectiveness in chronic diseases such as hypertension, neurodegenerative and oxidative conditions, and dyslipidemia. In acute infections, benzofuran revealed anti-infective properties against microorganisms like viruses, bacteria, and parasites. In recent years, the complex nature and the number of acquired or resistant cancer cases have been largely increasing. Benzofuran derivatives revealed potential anticancer activity with lower incidence or severity of adverse events normally encountered during chemotherapeutic treatments. This review discusses the structure–activity relationship (SAR) of several benzofuran derivatives in order to elucidate the possible substitution alternatives and structural requirements for a highly potent and selective anticancer activity.

## 1. Introduction

**Figure 1:**
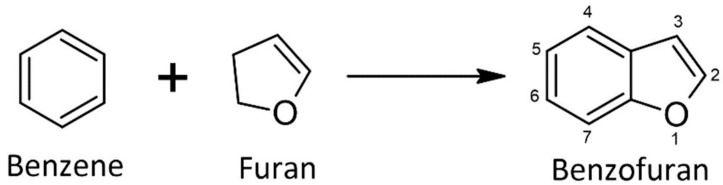
Chemical structure of benzofuran.

Several heterocyclic compounds are found in many medications and have formed an essential base for medicinal chemistry research. This is mainly due to heterocyclic compounds’ versatility and distinctive physicochemical features [1]. Among these discovered heterocyclic compounds is benzofuran [2], known as a natural compound originating from plants such as Asteraceae, Rutaceae, Liliaceae, and Cyperaceae [3]. Benzofurans can also emerge from non-natural sources through the dehydrogenation of 2-ethylphenol [4,5]. Structurally, benzofuran is characterized by a distinctive motif consisting of fused benzene and furan rings, as illustrated in Figure 1 [6]. 

It is suggested that introducing substituents at specified positions within the benzofuran’s core [2] results in new derivatives with unique structural characteristics that may possess an excellent therapeutic value [7]. Therefore, in recent years, derivatives of benzofurans have been frequently used in the development of new drugs [8]. These derivatives exhibited a promising anti-infective activity against bacteria, viruses, and parasites [9,10,11]. For example, in treating neurodegenerative disorders, derivatives of benzofurans revealed potential efficacy in slowing down the progression of Alzheimer’s [12] as well as minimizing Parkinson’s severity [13] and presented potential neuroprotective functions in brain disorders [14]. Furthermore, derivatives of benzofurans have the ability to achieve anti-dyslipidemic and antioxidative effects [15]. Some researchers extended the use of benzofurans’ derivatives to design an effective class of benzofuran-based vasodilators to treat some cardiovascular conditions [16]. In practice, the synthetic derivatives of benzofurans are represented by Amiodarone, which is used in the treatment of ventricular and supraventricular arrhythmias [17], and by Bufuralol as a non-specific β-adrenergic blocker with an affinity for β1- and β2-adrenergic receptors [18,19]. 

Despite the major progress that has been achieved in research, there are still barriers limiting the effective improvement of therapy, especially in cancer [20]. Nowadays, cancer is known to be the leading cause of death worldwide, accounting for approximately 10 million deaths in 2020 [20]. As cancer cases are constantly increasing, oncology research is investing significant efforts to identify novel, safe and effective therapies to minimize critical side effects caused by conventional treatments [19]. 

Fused heterocyclic ring systems have emerged as important scaffolds with many biological properties [1,21]. Accordingly, the peculiar structural motif of oxygen-containing heterocycles demonstrates a wide range of biological and pharmacological activities, including anticancer properties [2,18,22]. Earlier structure–activity relationship (SAR) studies of benzofurans’ derivatives found that ester or heterocyclic ring substitutions at the C-2 position were crucial for the compounds’ cytotoxic activity [18]. These modifications have a significant role in influencing the selectivity of these compounds toward cancer cells, which have significant importance given the damage to normal cells caused by the cytotoxic side effects of anticancer therapy. Therefore, this review will discuss the SAR of several anticancer derivatives of benzofurans to determine the critical substitution patterns and structural requirements useful to gain potent and selective anticancer activity.

## 2. Materials and Methods

The aim of this review is to highlight and discuss the contribution of benzofurans’ derivatives as anticancer agents. This review will discuss how the SAR of benzofuran can be used to predict their biological activity and better understand their applications in cancer treatment. 

A comprehensive electronic literature search of PubMed (MEDLINE), EMBASE, and Web of Science without language or date restrictions was conducted. The keywords related to “benzofuran” OR “derivatives” OR “compounds” OR “agent” OR “class” OR “anti-proliferative” OR “anti-tumor” OR “anti-cancer” OR “anti-neoplastic” OR “novel” OR “new” OR “active” OR “activity” OR “efficacy” OR “agent” OR “potent” OR “cytotoxic” OR “scaffolds” OR “heterocyclic” OR “modeling” OR “experimental” OR “computational” OR “potent” OR “selective” OR “drug design” OR “docking” OR “synthesis” OR “in vitro” were used to search the literature.

All figures in this paper were produced by the authors using ACD/ChemSketch, which is a free molecular modeling software used to create images of chemical structures.

## 3. Benzofuran Derivatives as Anticancer Agents

### 3.1. Halogenated Derivatives of Benzofuran

**Figure 2:**
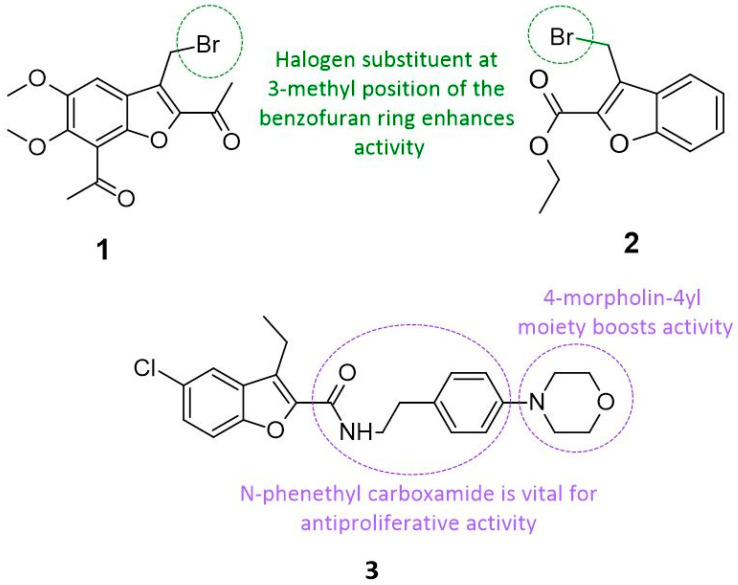
Chemical structure of halogenated derivatives of benzofuran **1–3**.

Some halogen additions into the benzofuran ring, such as bromine, chlorine, or fluorine atoms, have consistently resulted in a significant increase in anticancer activities [23,24,25,26,27,28,29,30]. This is most likely due to the ability of halogens to form a “halogen bond”; an attractive interaction between the electrophilic halogen and a molecule’s nucleophilic sites, which substantially improves the binding affinity [31,32]. For example, a set of seven derivatives (*1,1′*-*(5,6*-*dimethoxy*-*3*-*methyl*-*1*-*benzofuran*-*2,7*-*diyl) diethanone*) were synthesized via standard bromination reaction and condensation with aryl/heteroarylpiperazine [28]. Consequently, those novel halogen derivatives underwent *3*-*(4,5*-*dimethylthiazol*-*2*-*yl)*-*2,5*-*diphenyl*-*2H*-*tetrazolium bromide* (MTT) assays against three cancer cell lines (human chronic (K562), and acute (HL60) leukemia cells, human cervical cancer cells (HeLa)), and one normal endothelial cancer cell (HUVEC). Compound **1** (see Figure 2) has a bromine atom attached to the methyl group at the 3-position of the benzofuran ring; and was found to possess remarkable cytotoxic activity against K562, and HL60 leukemia cells with an inhibitory concentration (IC_50_) value of 5 μM and 0.1 μM (see Table 1), without cytotoxicity towards normal cells. This means that the position of the halogen in the benzofuran ring is a critical determinant of its biological activity [28].

In most cases, the halogen atom is attached to alkyl or acetyl chains rather than directly onto the benzofuran ring [28,33]. This placement does not deter the compound’s cytotoxic activity, as evidenced by electron-rich bromomethyl- or bromophenacyl-substituted benzofuran, which produced pronounced cytotoxic activity in both normal and cancer cells [34,35,36,37]. Selective Polo-like kinase 1 Polo-Box Domain (PLK1 PBD) inhibitor MCC1019 (compound **2**) (see Figure 2) is a bromomethyl-substituted benzofuran developed by Abdelfatah and colleagues for the treatment of lung cancer and evaluated via in silico, in vitro, and in vivo models [33]. There are two key interactions between MCC1019 and PLK1 at residues Tryptophan 414 (Trp414) and Histidine 538 (His538), which must be maintained for optimum activity. In vitro testing against lung adenocarcinoma cells (A549) showed that MCC1019 successfully inactivated the serine-threonine kinase (AKT) signaling pathway and inhibited cancerous cell replication, causing a mitotic catastrophe [33,38]. This resulted in achieving selective inhibition of PLK1 PBD with an IC_50_ of 16.4 μM (see Table 1) [33]. Further in vivo testing using a murine lung cancer model demonstrated a significant anticancer activity by reducing the growth of metastatic lesions in the lung without affecting body weight or vital organ size [33].

The substitution of the N-phenyl ring of the benzofuran with halogen is considered beneficial due to their hydrophobic and electron-donating nature, which enhances benzofuran’ cytotoxic properties [27]. Several studies in the literature have emphasized the influence of the position of the halogen atom on the cytotoxic activity [39,40]. So far, the maximum activities have been recorded when a halogen atom is placed at the para position of the N-phenyl ring [41]. 

A series of fourteen apoptotic anticancer derivatives were developed using the Allosteric cannabinoid receptor type 1 (CB1) modulator 5-chlorobenzofuran-2-carboxamides [42]. Each compound was then tested to evaluate its antiproliferative activity against the human mammary gland epithelial cell line (MCF-10A) via cell viability assays [43]. Although multiple compounds exhibited excellent antiproliferative activity against tumor cells, compound **3** stood out as the most active derivative (see Figure 2). According to the SAR analysis, the presence of the N-phenethyl carboxamide significantly enhances its antiproliferative activity. This activity was further enhanced by morpholinyl substitution at the para position of the N-phenethyl ring [42]. This explains why compound **3** exhibits similar antiproliferative activity to oral anticancer drug doxorubicin (IC_50_ of 1.136 μM) [42], (see Figure 2 and Table 1). Interestingly, regardless of the halogen used (e.g., Cl, Br, F, etc.), all the aforementioned halogen-substituted compounds exhibit significant cytotoxicity [28,33,42]. This suggests that, while the nature of the halogen does not impact the cytotoxic activity of the compound, the position of the halogen is of great importance [28].

### 3.2. Hybrid Benzofuran as Anticancer Agents

Recent studies have highlighted novel classes of hybrid benzofurans’ derivatives like chalcone, triazole, piperazine, and imidazole substituted benzofuran, which have emerged as potent cytotoxic agents [44,45,46,47,48,49]. Utilizing the synergetic cytotoxic effect of heteroatom-substituted benzofuran presents a promising approach for the development of potent anticancer drugs with activities against malignant tumors.

#### 3.2.1. Benzene-Sulfonamide-Based Benzofuran Derivatives

**Figure 3:**
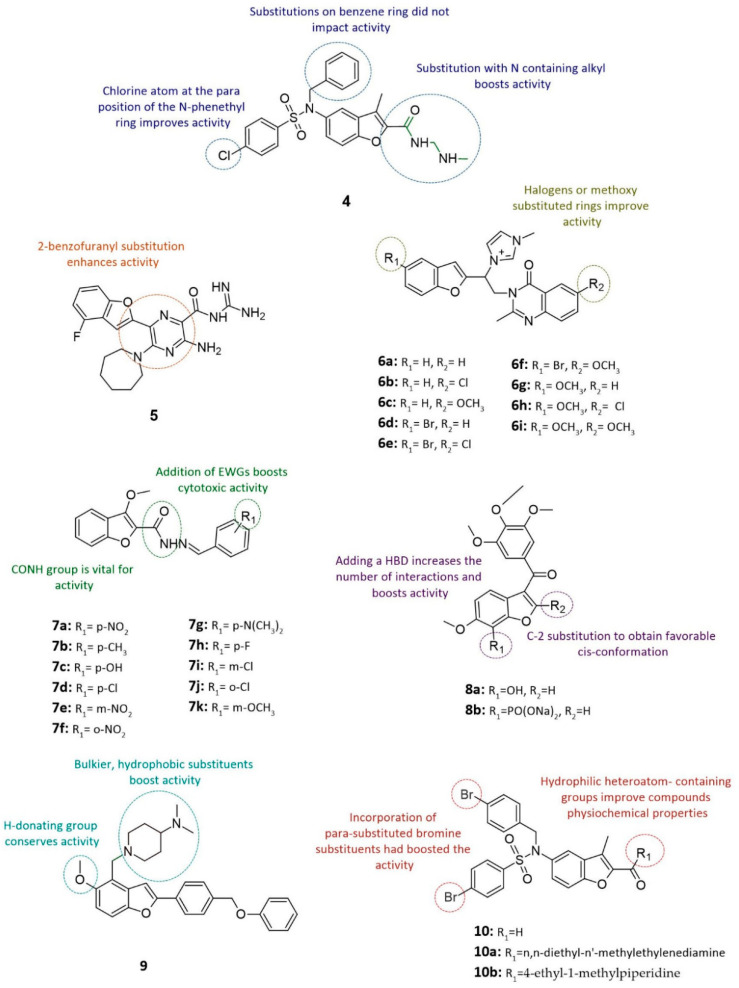
Chemical structures of anticancer hybrid benzofuran **4–10**.

Benzene-sulfonamide has become a biologically significant scaffold, with several of its derivatives being used as anticancer and antitumor agents [50]. Benzene-sulfonamide-based benzofuran derivative (*5*-*[benzyl*-*(4*-*chlorophenyl)sulfonylamino]*-*n*-*[2*-*(dimethylamino)ethyl]*-*3*-*methyl*-*1*-*benzofuran*-*2*-*carboxamide*) represented in compound **4** (see Figure 3) was designed and synthesized to inhibit the hypoxia-inducible factor (HIF-1) pathway [51], which is involved in the carcinogenesis of tumor protein (p53)-independent malignant cancers [51,52,53]. In vitro testing of compound **4** against HCT116 and HCT116−/− p53-null cell lines showed the inhibition of both p53-null cells and p53-mutated cells (see Figure 3 and Table 2). Thus, the addition of a chlorine atom at the para position along with the replacement of the ester group by N containing alkyl chains were major determinants for the antiproliferative activity [51].

#### 3.2.2. 6-Substituted Hexamethylene Amiloride (6-HMA)-Based Benzofuran Derivatives

The urokinase-type plasminogen activator (uPA) system mediates cancer invasion and metastasis through the uPA and its receptor (uPAR) [54]. Targeting uPA is one of the key strategies for combating metastasis in malignant cancers including triple-negative breast cancer [55,56]. In recent in vitro and in vivo studies, high doses of amiloride, a potassium channel blocker, have been found to inhibit uPA proteolytic activity, prompting the search for novel amiloride analogs as uPA inhibitors [57,58,59].

In order to investigate the potential of amiloride-benzofuran derivatives as uPA inhibitors, a series of 6-HMA, *6*-*N,N*-*(hexamethylene)* amiloride derivatives were synthesized via the Suzuki–Miyaura coupling reactions as potential uPA inhibitors [60]. The addition of a benzofuran group to 6-HMA yields a compound with higher potency, and cytotoxicity (Ki = 183 nM) [61]. In compound **5**, the addition of fluorine atom at position 4 of 2-benzofuranyl resulted in a 2-fold increase in potency and inhibitory activity (Ki = 88 nM; IC_50_ = 0.43 μM) [61] (see Figure 3 and Table 2). Such halogen substitutions at the para position of benzofuran are more likely to form favorable hydrophobic interactions, and therefore are more potent [62,63].

#### 3.2.3. Quinazolinone- and Imidazolium-Based Benzofuran Derivatives

Quinazolinone is an aromatic heterocyclic ring that contains a quinazoline with a carbonyl group [64]. Quinazolinone, like imidazole, is regarded by many as a privileged scaffold with significant anticancer properties [65]. Two of its derivatives, gefitinib, and erlotinib, were introduced to the market as anticancer agents [66]. One study reported the synthesis of a small library of benzofuran derivatives fused to two prominent scaffolds, imidazole and quinazolinone, to create a molecule with a desirable drug-like profile and cytotoxicity [47]. Accordingly, the cell viability and proliferation rates of nine hybrid derivatives (*1*-*[[(1*-*(benzofuran*-*2*-*yl)*-*2*-*(quinazolin*-*4(3H)*-*one*-*3*-*yl)]ethyl*-*1*-*yl]*-*3*-*methylimidazol*-*1*-*ium chloride*) or (compounds **6a–i**) were tested via MTT assays against human breast cancer (MCF-7) cells [47] (see Figure 3). All derivatives successfully inhibited the growth of cancer cells except compound **6e**. Analysis of its structural features suggested that the presence of two halogen-substituted rings coupled with the lack of methoxy substituent on the heterocyclic ring was detrimental to its activity, resulting in no cytotoxicity, as shown in Table 2. This is expected, as the addition of halogen-substituted rings is usually resulting compounds with little to no cytotoxic activity [47].

#### 3.2.4. Carbohydrazide- and Substituted Benzaldehydes-Based Benzofuran Derivatives

The condensation of 3-methyl-2-benzofuran carbohydrazide with various substituted benzaldehydes yielded a set of new benzofuran derivatives, compounds **7a–k** (see Figure 3). The eleven benzofuran analogues were screened for potential anticancer activity using the triphenyl blue dye exclusion technique on Erlich ascites carcinoma (EAC) cells [67,68,69]. Out of these eleven benzofuran analogues, derivatives **7a**, **7c**, **7d**, **7f**, **7i**, and **7j** demonstrated the greatest anticancer activity, as evidenced by their high cytotoxic concentration scores (CTC_50_) shown in Table 3. The SAR results have shown that the presence of the CONH group is necessary for anticancer activity [70]. The addition of phenol and chlorine groups in compounds **7c**, **7d**, and **7i** increased the number of binding interactions formed with the target, resulting in improved anticancer activity (see Figure 3). As for the nitro group in compound **7a**, it significantly boosted activity by reducing the melting temperature of DNA in EAC cells [71] (see Figure 3). Interestingly, the phenolic hydroxy group of benzofuran was found to be crucial for modulating anticancer activity. The presence of a hydrogen donating group promotes the formation of favorable interactions with the target, hence inducing its cytotoxic properties [17,44,70,72].

#### 3.2.5. Trimethoxyacetophenone-Based Benzofuran Derivatives

Combretastatin A-4 (CA-A4), which is a naturally occurring chemical, isolated from the roots of Combretum Caffrum, has recently attracted considerable attention for its antitumor and antimitotic activity [2,73]. The CA-A4 analogue (compound **8**) consists of trimethoxy acetophenone and a benzofuran core, and it has an IC_5O_ of 0.43 μM (see Figure 3). Subsequently, Flynn and colleagues used compound **8** as the lead compound for the SAR-guided design of novel tubulin polymerization inhibitors [74]. The results demonstrated that the introduction of C7-OH and a C2-substituent, as seen in compound **8a** (BNC105), improved its anticancer activity with a tubulin inhibition IC_50_ of 0.8 μM [74] (see Figure 3 and Table 2). Notably, the observed antimitotic activity is approximately tenfold stronger than that of the lead compound. The presence of a hydrogen bond donor (hydroxyl) at C7 adds to the pharmacophore’s interactions; as for the C-2 substituent, it maintains conformational bias, ensuring that the compound remains in the cis-conformation. Further efforts to enhance the activity were made by formulating a prodrug, disoduimphosphase ester derivative compound **8b** (BNC105P), which is rapidly cleaved in vivo to return to its active state compound **8a** [74] (see Figure 3). When tested in vitro, the prodrug produced tenfold stronger antitumor activity, eightyfold better selectivity, and a fivefold longer half-life than the free drug [74]. This means that adjusting the formulation is equally important to modifying the substituents on the compound in terms of increasing antitumor activity.

#### 3.2.6. N-Methylpiperidine-Based Benzofuran Derivatives

The hallmark of many cancers is the activation and dysregulation of the AKT/mammalian target of the rapamycin (mTOR) pathway, making it promising for drug discovery [75,76,77]. A series of mammalian targets of the rapamycin complex 1 (mTORC1) protein complex inhibitors were synthesized by performing different isosteric replacements on the lead compound *ChemBridge 5219657*, which was identified through high-throughput screening (HTS) [78,79]. Derivative *1*-*((2*-*(2*-*(benzyloxy) phenyl)*-*5*-*methoxybenzofuran*-*4*-*yl) methyl)*-*n, n*-*dimethylpiperidin*-*4*-*amine* (compound **9**) was found to exhibit the greatest cytotoxic activity against head and neck (SQ20B) cancer cell line with an IC_50_ value of 0.46 μM (see Figure 3 and Table 2). The replacement of the phenolic hydroxyl group with another H-bond donor like triflylamide conserved the cytotoxicity of the compound, whereas replacement with an H-bond acceptor altered its activity [78,79]. Whilst the introduction of triflate ester (a group that cannot donate or accept an H-bond) was well tolerated, the absolute removal of the phenolic hydroxy diminished the cytotoxicity of the compounds. Furthermore, substituting the dimethylamine and benzyl groups, with bulkier amine-containing groups such as 4-piperidino-piperidine, enhanced the cytotoxicity of the compounds [79]. 

Hypoxic microenvironments accelerate tumor metastasis and progression in solid tumor cancers, including pancreatic ductal adenocarcinoma (PADC) [80,81]. With the HIF-1 pathway being a target of interest, a small library of thirty-two benzofuran-derived HIF-1 inhibitors based on compound **10** were developed [82] (see Figure 3). MTT assays have found that derivatives 10a and 10b exhibit similar activity, but derivative *5*-*(4*-*bromo*-*N*-*(4*-*bromobenzyl) phenylsulfonamido)*-*3*-*methyl*-*N*-*(1*-*methylpiperidin*-*4*-*yl) benzofuran*-*2*-*carboxamide* (compound **10b**) emerged as the most promising candidate due to its significant antiproliferative activity and selective inhibition of HIF-1 pathway [83] (see Figure 3 and Table 2). The inclusion of hydrophilic heteroatom-containing groups, like piperidine, on the benzofuran ring significantly improved the compound’s physicochemical properties [82]. Additionally, the para-substituted halogen on the phenylsulfonyl- and N-containing alkyl chains contributed to the resultant antiproliferative activity [83]. 

**Table 2:** In vitro cytotoxicity of hybrid benzofuran derivatives **4**–**20** against multiple cancer cell lines.

Compound	Cell Line	IC_50_ (μM)	References
**4**	HCT116 (p53-null)	2.91	[51]
	MDA-MB-435s (p53-mutated)	4.71	
**5**	uPA	0.43	[61]
**6a**	MCF-7	7.70	[47]
**6b**	MCF-7	9.14	
**6c**	MCF-7	1.00	
**6d**	MCF-7	20.58	
**6e**	MCF-7	inactive	
**6f**	MCF-7	73.26	
**6g**	MCF-7	1.00	
**6h**	MCF-7	100	
**6i**	MCF-7	0.57	
**8**	Tubulin	0.43	[74]
**8a**	Tubulin	0.76	
**8b**	Tubulin	ND	
**9**	SQ20B	0.46	[79]
**10**	ND	ND	[82]
**10a**	PANC-1	1.52	
	BxPC3	1.08	
	HCT116	2.39	
	HCT116(p53−/−)	1.66	
	MCF-7	2.84	
	A549	2.98	
	MDA-MB-231	3.73	
**10b**	PANC-1	1.07	
	BxPC3	0.65	
	HCT116	1.81	
	HCT116(p53−/−)	1.61	
	MCF-7	2.39	
	A549	2.68	
	MDA-MB-231	1.90	
**11a**	A549	0.12	[84]
	Hela	26.32	
	SGC7901	2.75	
**11b**	A549	6.25	
	Hela	18.71	
	SGC7901	36.23	
**11c**	A549	8.11	
	Hela	28.74	
	SGC7901	>40	
**11d**	A549	34.13	
	Hela	12.68	
	SGC7901	7.45	
**12**	HT-1080	8.86	[85]
**13a**	HL60	2.34	[86]
	SMMC-7721	2.63	
	A549	4.5	
	MCF-7	3.24	
	SW480	3.61	
**13b**	HL60	0.64	
	SMMC-7721	2.10	
	A549	3.34	
	MCF-7	4.78	
	SW480	5.56	
**13c**	HL60	0.61	[86]
	SMMC-7721	2.30	
	A549	5.35	
	MCF-7	3.03	
	SW480	3.14	
**13d**	HL60	0.08	
	SMMC-7721	0.52	
	A549	0.55	
	MCF-7	0.51	
	SW480	0.47	
**14a**	ND	ND	[87]
**14b**	ND	ND	
**14c**	ND	ND	
**14d**	ND	ND	
**15a**	MCF-7	1.90	[88]
	A549	2.38	
	Colo-205	2.11	
	A2780	1.05	
**15b**	MCF-7	3.90	
	A549	4.17	
	Colo-205	ND	
	A2780	ND	
**15c**	MCF-7	0.011	
	A549	0.073	
	Colo-205	0.10	
	A2780	0.034	
**15d**	MCF-7	7.23	
	A549	6.91	
	Colo-205	2.84	
	A2780	10.2	
**15e**	MCF-7	12.5	
	A549	5.34	
	Colo-205	ND	
	A2780	9.55	
**15f**	MCF-7	3.16	
	A549	ND	
	Colo-205	7.10	
	A2780	8.64	
**15g**	MCF-7	10.76	
	A549	19.42	
	Colo-205	ND	
	A2780	ND	
**15h**	MCF-7	1.55	
	A549	1.93	
	Colo-205	1.28	
	A2780	2.13	
**15i**	MCF-7	0.21	
	A549	0.43	
	Colo-205	0.17	
	A2780	1.84	
**15j**	MCF-7	0.14	
	A549	0.25	
	Colo-205	0.12	
	A2780	0.33	
**16**	K562	ND	[89]
**17a**	K562	ND	
**17b**	K562	ND	
**18**	A549	9	[90]
	MCF-7	2	
	PC-3	10	
**19**	A549	6.3	[49]
**20a**	A549	10.9	
**20b**	A549	Inactive	

The definitions of all abbreviations are provided in a list at the end of the manuscript.

**Table 3:** In vitro cytotoxicity inhibition of hybrid benzofuran derivatives **7a**–**k** against EAC cancer cell lines.

Compound	CTC_50_ (μM/mL)	Reference
**7a**	35.5	[71]
**7b**	472	
**7c**	33.5	
**7d**	33.75	
**7e**	255	
**7f**	43	
**7g**	280	
**7h**	365	
**7i**	34	
**7j**	49	
**7k**	478	

The definitions of all abbreviations are provided in a list at the end of the manuscript.

#### 3.2.7. Piperazine-Based Benzofuran Derivatives

**Figure 4:**
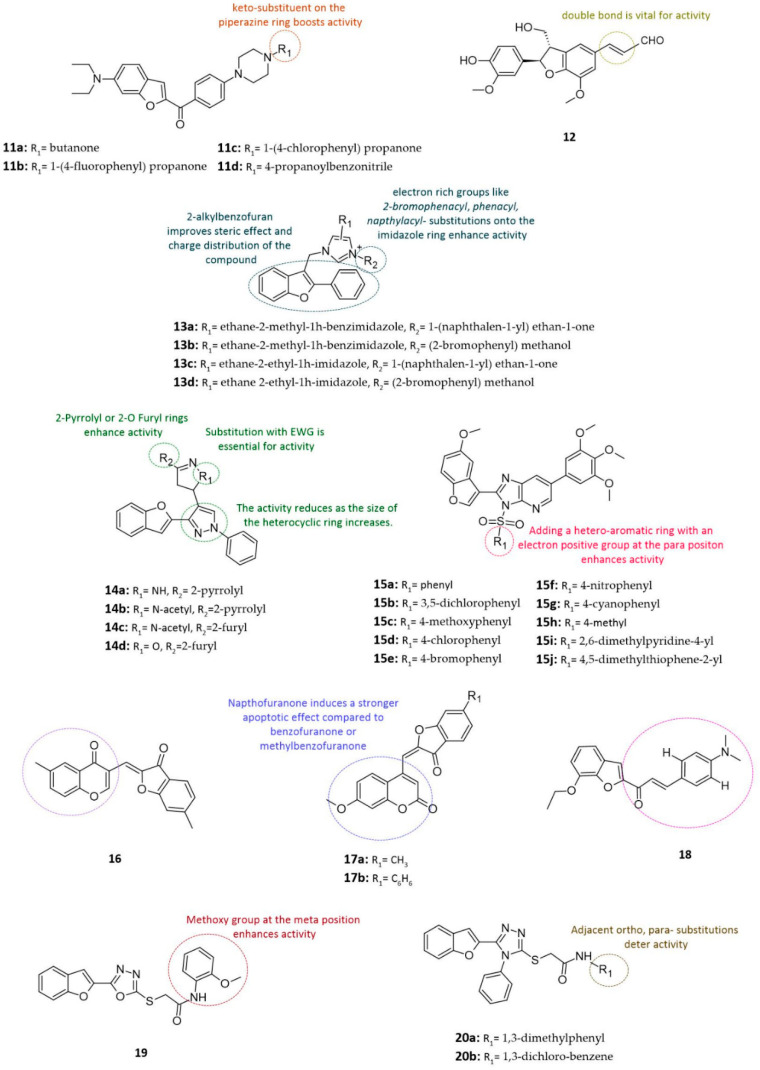
Chemical structures of anticancer hybrid benzofuran **11–20**.

Piperazine is a six-membered ring containing two nitrogen atoms at opposite positions [91]. In vitro and/or in vivo studies have shown that several piperazine compounds revealed significant activities against a variety of cancers cell lines [92]. Given this, a hybrid of 2-benzoyl benzofuran with N-aryl piperazine linker is considered to be more biologically active than unsubstituted benzofuran [18,40,84]. Benzofuran piperazine hybrids were designed, synthesized, and tested via MTT assays against lung cancer (A549), human cervical carcinoma (Hela), and colonic cancer (SGC7901) cell lines [84]. Derivatives bearing keto-substituent on the piperazine ring (compounds **11a–d**) exhibited the most cytotoxic activity against cancer cells [84] (see Figure 4). Similarly, the addition of an electron-withdrawing group or halide such as fluoro-, chloro-, and cyano- at the para position of benzene in compounds **11b**, **11c**, and **11d** was beneficial for anticancer activity [84] (see Figure 4). Furthermore, compound **11a** showed promising activity and selectivity to lung (A549) and colonic cancer (SGC7901) cell lines with IC_50_ values of 0.12 μM and 2.75 μM, respectively [84] (see Figure 4 and Table 2). 

#### 3.2.8. Neolignans-Based Benzofuran Derivatives

Naturally occurring dihydrobenzofuran neolignans are often found in high concentrations in aerial parts of plants like Mappianthus iodoies, Dorstenia kameruniana, and Aristolochia fordiana [45,93,94,95,96,97]. Many neolignans have shown considerable activity against a variety of cancers cell lines [98]. Neolignan-based benzofurans’ derivatives are expected to benefit from the synergistic cytotoxic effect of both molecules. Thus, eight dihydro benzofuran neolignans analogs were isolated from the seeds of crataegus pinnatifida [85]. In vitro testing recognized *7R,8S*-*balanophonin* (compound **12**) as the most potent analogue, with stronger inhibitory activity against HT-1080 cancer cells than positive control 5-fluorouracil (5FU) (IC_50_ = 35.62 μM) (see Figure 4 and Table 2). The SAR studies of hybrid dihydrobenzofuran neolignans revealed that the presence of a double bond at C-7′/C-8′ next to the aromatic ring is vital for cytotoxicity and that the reduction of the double bond can reduce the activity by tenfold or greater [85]. 

#### 3.2.9. Imidazole-Based Benzofuran Derivatives

Imidazoles are five-membered, nitrogen-containing heterocycles with significant anticancer activity against a variety of biological targets [99]. However, there is no consensus surrounding the cytotoxic activity of benzofuran-imidazole derivatives [48,100,101]. It has been reported that the addition of an imidazole ring to the benzofuran produced compounds with weak cytotoxic properties [36]. Therefore, to yield optimal benzofuran imidazole hybrids, some modifications must be implemented. The 2-benzylbenzofuran ring is altered to 2-alkylbenzofuran to improve both the steric effect and charge distribution of the compound [100,101]. Then, electron-rich groups like 2-bromophenacyl, phenacyl, and napthylacyl- are substituted onto the imidazole ring, preferably into the 3-positon [102]. These alterations are crucial to ensure a maximal cytotoxic activity against cancer cells. Similar findings were observed in 2-phenyl-3-alkylbenzofuran imidazole/triazole hybrids (compounds **13a–d**) (see Figure 4) [86]. These highly potent anticancer derivatives often include a 2-ethyl-imidazole or benzimidazole ring with a 2-bromobenzyl or napthylacyl substituent at the 3-position of the imidazole ring, all of which are important groups in modulating antitumor activity [103]. Among these compounds, compound **13d** has shown the strongest inhibitory activity and selectivity towards breast cancer (MCF-7) and colon cancer (SW480) cells, with IC_50_ values ranging from 0.08 to 0.55 μM [86] (see Figure 4 and Table 2). 

#### 3.2.10. Pyrazole-Based Benzofuran Derivatives

The non-receptor tyrosine kinase (c-Src) has been identified as a promising target for cancer treatment, sparking the interest of researchers [104,105,106]. Pyrazole is a five-membered aromatic heterocyclic ring containing two neighboring nitrogen atoms [107]. Pyrazole derivatives have previously demonstrated antitumor activity against numerous types of cancer [108]. In an effort to discover novel potent c-Src inhibitors as anticancer agents, a set of benzofuran-pyrazoles hybrids containing chalcones, pyrazoline, isoxazole, and thiopyrimidine substituents were in vitro-synthesized and tested for their anticancer activity [87].

Compounds **14c** and **14d**, which consist of *3*-*furano*-*N*-*acetylpyrazoline* and *3*-*furano*-*isoxazole* rings, respectively, exhibited remarkable and broad-spectrum anticancer activity (see Figure 4). Incorporating acetyl, an electron-withdrawing group, into the N-1 of the pyrazoline ring appears to be essential for antiproliferative activity. Hence, the derivatives lacking the acetyl group such as compound **14a** exhibited weak anticancer activity in-vitro [87] (see Figure 4). Increasing the size of the hetero-ring systems attached to the parent core resulted in weak-to-moderate antiproliferative potency [21]. Among all derivatives, compound **14b** containing *3*-*pyrrolo*-*N*-*acetylpyrazoline* demonstrated significant antiproliferative and anticancer activity against leukemia, lung cancer, colon cancer, central nervous system (CNS) cancer, melanoma, ovarian cancer, breast cancer, and renal cancer cells [87] (see Figure 4). Enzyme assays of compound **14c** detected significant inhibition of Src and zeta-chain-associated protein (ZAP-70) kinases [87] (see Figure 4). Overall, the potent antitumor activity and favorable absorption, distribution, metabolism, and excretion (ADME) characteristics of compound **14b** make it a viable candidate worthy of further investigation and modifications (Figure 4). 

#### 3.2.11. Imidazopyridine-Based Benzofuran Derivatives

Imidazopyridine is fused bicyclic heterocycles that are synthesized by several strategies such as condensation, oxidative coupling, tandem reaction, etc. [109]. A series of imidazopyridine-substituted benzofurans (compounds **15a–j**) were derived from sulfonamides, and subsequently underwent MTT assays to evaluate their in vitro cytotoxicity against human cancer cells [110] (see Figure 4). Moreover, compounds **15a**, **15c**, **15h**, **15i**, and **15j** were found to produce considerable anticancer activity against tested cell lines [88] (see Figure 4). Among these, derivative **15c**, with the greatest cytotoxicity, significantly inhibited the growth of breast (MCF-7), lung (A549), colon (Colo-205), and ovarian (A2780) cancer cell lines with IC_50_ values of 0.011, 0.073, 0.10, and 0.034 μM [88], respectively (see Figure 4 and Table 2). The SAR has shown that the addition of electron-positive groups at the para position on the phenyl group significantly improved anticancer activity, regardless if it’s a strong group like 4-methoxy (compound **15c**) or a weak group like 4-methyl (compound **15h**). On the other hand, substitution with electron-withdrawing groups like chloro (**15d**), bromo (**15e**), nitro (**15f**), and cyano (**15g**) resulted in significant drop-in activity (Figure 4 and Table 2). Interestingly, compound **15a** lacked any phenyl ring substituents but still maintained good anticancer activity [88] (see Figure 4). Furthermore, replacing the aryl ring with a hetero-aromatic ring such as *2,6*-*dimethylpyridine* (compound **15i**) or *4,5*-*dimethylthiophene* (compound **15j**) rings was more beneficial for anticancer activity than keeping the aryl ring (compound **15a** and **15h**) [88] (see Figure 4).

#### 3.2.12. Aurones-Chromone- and -Coumarin-Based Benzofuran Derivatives

Flavonoids, aurones, chromones, and coumarins are abundantly found in plants, fungi, and bacteria [109]. These natural products are capable of modulating a wide range of biological pathways and achieving selective anticancer activity with few side effects [109,111,112]. Yet, only a limited number of hybrids with aurone-chromone, -coumarin fused heterocycles have been reported. Therefore, a series of 26 hybrid compounds between benzofuran core of aurones-chromone and -coumarin were designed [89]. This combination takes advantage of the potential synergistic anticancer effect of these flavonoids [113,114,115]. These derivatives were then evaluated for their anticancer activity against a panel of human leukemia cells (K562) at different concentrations. In particular, compounds **16**, **17a**, and **17b** were able to induce around 24% apoptosis [89] (see Figure 4). Interestingly, the potency of the compounds is unaffected by different substitutions of the chromone [29]. Furthermore, it appears that exchanging the benzofuranone or methylbenzofuranone moieties with napthofuranone induces a stronger apoptotic effect [89]. In order to understand the pro-apoptotic properties of these benzofuran–coumarin derivatives, *(Z)*-*7*-*methoxy*-*4*-*[(6*-*methyl*-*3*-*oxobenzofuran*-*2(3H)*-*ylidene) methyl]*-*2H*-*coumarine* (compound **17a**) was compared to *7*-*methoxy*-*coumarin*-*4*-*aldehyde and (Z)*-*2*-*(4*-*methoxybenzylidene)*-*6*-*methylbenzofuran*-*3(2H)*-*one* by testing them in K526 cells at doses ranging from 5 to 100 μM [89]. The results demonstrated that compound **17a** produced the strongest apoptosis induction at higher doses, outperforming both unsubstituted benzofuran and coumarins [89]. These findings imply that coupling aurone-like benzofuran with a chromone or coumarin can yield novel compounds with more potent pro-apoptotic properties compared to unconjugated benzofuran.

#### 3.2.13. Chalcone-Based Benzofuran Derivatives

Many naturally occurring compounds are derived from plants, including the simple chalcone scaffold [116]. These structures are simple to synthesize, allowing for the chalcones to be incorporated into several derivatives with a wide range of biological activities [117]. Moreover, chalcones have been recognized as a valuable scaffold with potent anticancer activity [118]. Thus, a synergistic cytotoxic effect could be observed after the hybridization of chalcones and benzofuran, yielding compounds that are used to treat malignant tumors [18,29,44].

Encouraged by the anticancer potential of chalcones, a set of *1*-*(7*-*ethoxy*-*1*-*benzofuran*-*2*-*yl)* substituted chalcone derivatives via the base-catalyzed Claisen-Schmidt reaction was synthesized [46,90]. All derivatives were then tested by sulforhodamine B (SRB) and adenosine 5′-triphosphate (ATP) cell viability assays, against breast (MCF-7), non-small-cell lung (A549), and prostate (PC-3) cancer cell lines [43]. The best cytotoxic activity was observed in chalcone derivative compound **18**, with IC_50_ values ranging from 2 to 10 μM [90] (see Figure 4 and Table 2). Interestingly, compound **18** showed selective cytotoxicity toward human breast cancer cell line (MC-7), while being non-toxic towards normal breast cancer cells (MRC5). Furthermore, compound **18** was successful in inducing apoptosis in cancer cells while maintaining a promising safety profile, indicating that hybrid benzofuran chalcones have greater cytotoxic activity compared to unsubstituted benzofuran [90].

#### 3.2.14. Oxadiazole- and Triazole-Based Benzofuran Derivatives

Oxadiazoles and triazoles are nitrogen-oxygen and nitrogen-containing five-membered heterocyclic aromatic rings commonly hybridized with other anticancer scaffolds, such as benzofuran [119,120,121]. These hybrid derivatives have shown substantial anticancer potential and play essential roles in cancer management [122,123]. Hence, ultrasound- and microwave-assisted green synthetic protocols were implemented for synthesizing a set of 15 benzofurans–oxadiazole and –triazole. Then, those compounds were evaluated for the anticancer activity against the lung cancer cell line (A549) [49]. Compound **19**, benzofuran-oxadiazole hybrid, was reported as the most potent anticancer derivative, with cell viability of 27.49 μM and IC_50_ of 6.3 μM, outperforming reference drugs crizotinib and cisplatin, which had IC_50_ of 8.54 and 3.88 μM, respectively [49]. The enhanced anticancer activity is believed to be due to meta methoxy or para ethoxy substitutions on the phenyl ring of N-(substituted-phenyl)-acetamide (see Figure 4). 

Although benzofuran triazole derivatives 20a and b exhibit excellent thrombolysis activity and minimal toxicity, they did not demonstrate strong anticancer activity against A549 cancer cells (see Figure 4 and Table 2). The presence of two adjacent electron-withdrawing chloro groups at the ortho and para positions of the phenyl ring in compound **20a** was detrimental to its anticancer activity [49]. Similarly, in compound **20b**, the addition of two adjacent methyl groups on the ortho and para positions of the phenyl ring yielded an inactive compound [49]. These SAR studies highlight the possible positive and negative impacts of structural modifications to oxadiazole- and triazole-benzofuran derivatives as anticancer drug candidates.

### 3.3. Cytotoxicity of Benzofurans’ Derivatives against Selected Cancer Cell Lines

Many of the compounds presented have been tested against the same cancer cell lines, and while they show high cytotoxicity, it is notable how different substitutions can influence the compound’s cytotoxicity against selected cancer cell lines. Imidazopyridine-benzofuran analogs bearing electron-positive groups at the 4-position on the phenyl group, for example, have significantly improved anticancer activity against various cancer cell lines (A549, MCF-7, HL-60, SW480, A2780, and Colo-205) [88]. The most effective modifications to the cytotoxicity of MCF-7 cell lines were quinazolinone and Imidazolium, Imidazole, and Chalcone-based benzofuran compounds [47,86,90]. A halogen atom attached to the methyl group at the 3-position of the benzofuran ring promotes cytotoxicity toward both A549 and HL60 cell lines [28,33]. More specifically, the presence of oxadiazole and triazole-benzofuran hybrids further boosts the anticancer activity of A549 cells [49]. The majority of the novel hybrids demonstrated potential anticancer agents against specific cancer cell lines, while maintaining a remarkable safety profile against normal cells. Most of the novel hybrids demonstrated potential anticancer agents against specific cancer cell lines while maintaining a remarkable safety profile against normal cells. Hence, benzofuran derivatives have the potential to be developed as novel therapeutic agents given their recent experimental findings and documented selectivity against cancer cells.

## 4. Conclusions

This review suggests benzofuran as a versatile scaffold with significant anticancer activity on various human cells such as breast, lung, and prostate cancer. Understanding the SAR of benzofurans’ derivatives facilitates the design and development of novel, safe, and potent in vitro therapeutic options in cancer. Therefore, this may provide a more robust assessment of anticancer activities before considering in vivo studies. The anticancer activity of benzofuran scaffolds is dependent on the type of substituent present and is frequently multifactorial. Furthermore, hybrid structures bearing benzofuran moiety stand out as highly potent anticancer agents. They utilize the functionalization or structural configuration of the conjugate molecule. This review recommends that studying the chemical structure of these compounds will result in anticancer agents that limit tumor progression with minimal adverse effects. Therefore, this could potentially have an impact on improving patients’ adherence to medication and subsequently disease prognosis.

## Figures and Tables

**Table 1:** In vitro inhibitory activities of halogenated derivatives of benzofuran against multiple cancer cell lines.

Compound	Cell Line	IC_50_, μM	GI_50_, μM	Reference
**1**	K562	5	ND	[28]
	HL60	0.1	ND	
**2**	PLK1 PBD	16.4	ND	[33]
**3**	A-549	ND	1.8	[42]
	MCF-7	ND	0.7	
	Panc-1	ND	1.3	
	HT-29	ND	1.6	

The definitions of all abbreviations are provided in a list at the end of the manuscript.

## Abbreviations

5FU	5-fluorouracil
6-HMA	6-substituted hexamethylene amiloride
6-HMA	6- N, N-hexamethylene
ADME	Absorption, distribution, metabolism, and excretion
AKT signaling	Serine–threonine kinase signaling
ATP	Adenosine 5′-triphosphate
A549	Hypotriploid alveolar basal epithelial cell lines
A2780	Ovarian cancer cell line
BNC105P	Disoduimphosphase ester derivative compound 8b
BxPC3	Human pancreatic cancer cell lines
CB1	5-chlorobenzofuran-2-carboxamides
CNS	Central nervous system
CB1 modulator	Cannabinoid receptor type 1 modulator
CA-A4	Combretastatin A-4
CTC50	cytotoxic concentration scores
Colo-205	Colon cancer cell lines
EAC	Erlich ascites carcinoma cells
HT-29	Human colorectal adenocarcinoma cell lines
HCT116	Human colorectal carcinoma cell lines
HIF pathway	Hypoxia-inducible factor pathway
HUVEC	normal endothelial cancer cell lines
HT-1080	Fibrosarcoma cell lines
HL60	Human acute leukemia cells
HeLa	human cervical cancer cells
HTS	High-Throughput Screening
IC50	half-maximal inhibitory concentration
Ki	Dissociation constant
K562	Human leukemia cell lines
MCF-7	human breast cancer cells
MTT	3-(4,5-dimethylthiazol-2-yl)-2,5-diphenyl-2H-tetrazolium bromide
mTOR pathway	mammalian target of the rapamycin
mTORC1	Mammalian target of rapamycin complex 1
MDA-MB-23	Metastatic adenocarcinoma cell lines
MCF-10A	human mammary gland epithelial cell line
MRC5	normal breast cancer cells
NA	Not Applicable
Panc-1	Human pancreatic cancer cell lines
P53	Tumor protein
PADC	pancreatic ductal adenocarcinoma cell lines
PC-3	Prostate cancer cell lines
PLK1 PBD inhibitor	Polo-like kinase 1 Polo-Box Domain inhibitor
SAR	Structure–activity relationship
SRB	sulforhodamine B
SQ20B	head and neck cancer cell lines
SMMC-7721	Hepatocellular carcinoma cell lines
SGC7901	Colonic cancer cell lines
Src	Non-receptor tyrosine kinase protein
SW480	Colon cancer cell lines
TNBC	Triple-negative breast cancer cell lines
uPA	urokinase-type plasminogen activator
uPAR	urokinase-type plasminogen activator receptor
ZAP-70 kinases	Zeta-chain-associated protein kinase 70
